# Sleep, Stress, and Cardiometabolic Health in Women of Childbearing Age with Overweight and Obesity

**DOI:** 10.1089/whr.2023.0138

**Published:** 2024-02-21

**Authors:** Sarah S. Farabi, Cindy Schwarz, Alicia Persaud, Amanda Gilbert, Debra Haire-Joshu, Rachel G. Tabak

**Affiliations:** ^1^ Office of Nursing Research, Goldfarb School of Nursing, St. Louis, Missouri, USA.; ^2^ Division of Nutritional Science & Obesity Medicine, Center of Human Nutrition, Washington University in St. Louis School of Medicine, St. Louis, Missouri, USA.; ^3^ Brown School, Washington University in St. Louis, St. Louis, Missouri, USA.; ^4^ Public Health at the Brown School, Washington University in St. Louis, St. Louis, Missouri, USA.; ^5^ Department of Medicine, School of Medicine, Washington University in St. Louis, St. Louis, Missouri, USA.

**Keywords:** sleep, basic needs, obesity, stress, women of childbearing age

## Abstract

**Background:**

Sleep is important for health, but its relationship to cardiometabolic health in women of childbearing age remains unclear. Furthermore, stress, unmet basic needs, and lack of physical activity may be related to disrupted sleep and poor cardiometabolic health in women of childbearing age and these relationships may differ by ethnicity. The purposes of this study were to investigate the relationship between sleep, markers of cardiometabolic health, stress, unmet basic needs, and physical activity in women of childbearing age with overweight or obesity and identify if these relationships differed between women that identified as Latino/Hispanic and non-Latino/Hispanic ethnicity.

**Methods:**

A secondary cross-sectional analysis was conducted using baseline data from a trial that embeds healthy eating and activity into a national home visiting program, Parents as Teachers. The sample was stratified based on self-reported ethnicity (Hispanic/Latino or non-Hispanic/Latino). Pearson's and Spearman's correlations were used to determine bivariate relationships among sleep, cardiometabolic variables, stress, unmet basic needs, and physical activity.

**Results:**

Two hundred seventy-six women, 46% of whom identified as Hispanic/Latino, were included in the analysis. Body mass index (BMI) was significantly correlated with sleep disturbance (ρ = 0.23, *p* = 0.01) in women who identify as Hispanic/Latino. Stress was positively related to sleep disturbance, sleep duration, and unmet needs for both groups of women. BMI was correlated with unmet basic needs in women who identified as non-Hispanic/Latino (ρ = 0.25, *p* = 0.01).

**Conclusions:**

Our results suggest that sleep, stress, and basic needs are important in understanding cardiometabolic health in women of childbearing age and these relationships differ depending on ethnicity. Clinical Trial Registration Number: NCT03758638.

## Introduction

Forty percent of women of childbearing age (18–40 years) report poor sleep^1^ and racial/ethnic disparities in sleep health have been reported among women in the United States.^2–4^ For example, shorter sleep duration, more variability in sleep timing, and less slow wave sleep (restorative sleep) have been reported in people who identify as Hispanic/Latino as compared with non-Hispanic White people.^5–7^ This is concerning as sleep is increasingly recognized as an important component of health.^8^ For women of lower socioeconomic status, some of these factors may contribute more heavily to disrupted sleep.

High levels of stress are associated with disrupted sleep.^9^ Women of childbearing age report high levels of stress.^10^ Higher levels of unmet basic needs are also related to higher stress levels in vulnerable (*i.e.*, low socioeconomic status [SES]) populations.^11^ However, it remains unclear if high levels of stress and unmet basic needs are related to poor sleep, particularly in women of childbearing age, and if this relationship is different depending on the women's self-identified ethnicity.

Physical activity has clear benefits on cardiometabolic health,^12^ but its relationship to sleep is less understood. There are some studies suggesting physical activity is related to shorter sleep duration,^13^ but others have suggested that sleep quality is higher with more physical activity.^13,14^ However, the relationship between sleep and physical activity has not been thoroughly investigated in women of childbearing age.

Poor sleep has been linked to decreased cardiometabolic health (*i.e.*, body mass index [BMI] and blood pressure).^8^ However, the relationship between sleep and cardiometabolic risk in women of childbearing age, especially women who identify as Hispanic or Latino (Hispanic/Latino) has not been widely investigated.^1^ Understanding the relationship between sleep and cardiometabolic risk in women of childbearing age is important as sleep is a potentially modifiable behavior. Furthermore, understanding factors that are related to poor sleep in women of childbearing age can help to inform interventions surrounding sleep health in this population.

The aims of this study were to (1) investigate the relationship between sleep and markers of cardiometabolic health (BMI and blood pressure) in women of childbearing age with overweight or obesity; (2) determine the relationship between stress, unmet basic needs, physical activity, and sleep in women of childbearing age with overweight or obesity; and (3) identify if these relationships differed by self-reported ethnicity (Hispanic/Latino or non-Hispanic/Latino).

## Materials and Methods

This is a secondary analysis of baseline data collected for a larger study, the details of methods have been previously published.^15^ In brief, from March 2019 to June 2022, women were recruited to participate in a cluster randomized trial of a lifestyle modification program (Healthy Eating and Active Living at Home), which embeds healthy eating and activity content into Parents as Teachers (PAT). PAT is a national home-visiting organization serving families prenatal through kindergarten (https://parentsasteachers.org).

Women were included if they were 18–45 years old, had overweight or obesity (BMI 25–45 kg/m^2^), participating or willing to participate in PAT, and spoke English or Spanish. All study procedures were approved by the Institutional Review Board at Washington University in St. Louis. At baseline, height and weight were collected and surveys were completed regarding demographics, health behaviors, and basic needs. Surveys were completed either on an iPad in person (March 2019 to March 2020) or over the phone (after the start of the COVID pandemic in March 2020). Data collection was completed in English or Spanish depending on the preference of the participants.

### Demographic information

Questions were asked *via* survey about number of children in the home, highest grade completed, currently employed for wages/salary (yes/no), participation in assistance programs (Women, Infants, and Children [WIC], Supplementary Nutritional Assistance Program [SNAP], or other), and family history of diabetes or high blood pressure (yes/no or unsure). Ethnicity was collected *via* self-report and individuals selected if they identified as Hispanic or Latino (Hispanic/Latino) or non-Hispanic or Latino (non-Hispanic/Latino).

For purposes of this study, individuals who did not report ethnicity (*n* = 32) were not included in the analyses. These individuals did not differ from the study sample in BMI, age, or blood pressure (data not shown). Race was collected *via* self-report and individuals selected if they identified as American Indian or Alaskan, Asian, Native Hawaiian or Other Pacific Islander, Black or African American, White, or prefer not to say.

### Cardiometabolic variables

To calculate BMI, height was obtained *via* self-report, and weight was obtained using an electronic scale (Seca 874 dr). The average of two weights was taken. Participants were asked and remove shoes and excess clothing before stepping on the scale. Blood pressure was measured in accordance with the American Heart Association guidelines.^16^ Weight, height, and blood pressure were collected in-person until March 2020 and then collected remotely *via* phone or video call.

### Sleep, stress, and physical activity

Sleep was assessed using questions from the Sleep Heart Health Study questionnaire.^17^ This included a question on the duration of nightly sleep [*i.e.*, “On average, how many hours of sleep do you usually get at night (or your main sleep period)?”] and seven questions on sleep disturbance (trouble falling asleep, trouble staying asleep, wake up early, feel unrested during the day, excessively sleepy during the day, do not get enough sleep, and take sleeping pills to help sleep).

Responses to sleep disturbance questions had a 5-point Likert scale (0 = never, 1 = rarely, 2 = sometimes, 3 = often, and 4 = almost always). Each sleep disturbance question was then collapsed into two categories: infrequent, 0 (never, rarely, or sometimes), and frequent, 1 (often or almost always). The sum of the responses was then calculated to develop a sleep disturbance index which ranged from 0 to 7. Higher values indicated higher sleep disturbance. Stress was measured using the perceived stress scale-4.^18^

Scores range from 0 to 16, with higher scores reflecting higher stress levels. Physical activity was measured using the International Physical Activity Questionnaire.^19^ Physical activity levels are broken into low, moderate, and high categories. The high physical activity category indicated that the individual spends an hour or more a day conducting activity that is moderate intensity or higher. Moderate indicated the individual spends half an hour a day conducting moderate physical activity. Low indicated that the individual did not meet the criteria for moderate or high categories.

### Basic needs

Food insecurity was measured by asking if participants had worried in the past 12 months whether food would run out before getting money to buy more and if the food they bought did not last and they did not have money to get more.^20–22^ Responses were *often true, sometimes true, or never true.* If participants answered often or sometimes true to either question, they were considered food insecure. Questions to assess basic needs of housing stability, the ability to pay for necessities, bills, and unexpected expenses were added in January of 2020.^23^

Responses to these questions were on the same Likert scale (1 = Very likely, 2 = Likely, 3 = Unlikely, 4 = Very unlikely, and 5 = Don't know/not sure). These were recoded to “very likely” and “likely” as 0 or “yes” meaning this basic need is met, and “unlikely,” “very unlikely,” and “don't know/not sure” as 1 or “no” as this need is unmet. A basic needs variable was then created by adding the sum of these four variables, those with a sum of “0” indicated that their basic needs were met and ≥1 indicated that their needs were not met.

### Statistical analysis

Descriptive statistics were calculated for all variables for the entire group, and then by ethnicity. Depending on normality and variable characteristics, variables were compared using unpaired *t*-tests, Mann–Whitney *U* tests, or chi-square. Statistical significance was considered to be achieved at a *p*-value of <0.05. Pearson product correlations or Spearman correlations (ρ) were used to determine bivariate relationships between variables within each ethnicity (women identifying as non-Hispanic or Hispanic/Latino, regardless of race). Statistical analyses were performed by using STATA/IC 16.0 for Windows (STATA Corp LLC, TX).

## Results

A total of 276 participants were included in the full analysis, 46% of whom identified as Hispanic or Latino (Table 1). The average age of the sample was 30.8 ± 5.8 (mean ± standard deviation) years, and this did not differ between women who identified as Hispanic/Latino (30.2 ± 5.8 years) and women who identified as non-Hispanic/Latino (31.3 ± 5.7 years). Self-reported racial breakdown is included in Table 1. Since the basic needs questions were added later in the study, only a total of 174 women answered these questions, 42% of whom identified as Hispanic/Latino.

**Table 1.** Participant Demographics, Cardiometabolic Characteristics & Health Behaviors

	Entire cohort	Hispanic/Latino	Non-Hispanic	*p*
Cardiometabolic variables				
BMI (*n* = 276), mean (SD)	33.1 (5.5)	31.8 (4.9)	34.2 (5.7)	<0.001
SBP (*n* = 256), mean (SD)	113.4 (13.5)	109.2 (12.2)	116.8 (13.6)	<0.001
DBP (*n* = 256), mean (SD)	73.2 (11.4)	69.3 (10.7)	76.4 (10.9)	<0.001
Sleep				
Sleep duration (*n* = 257), hours	6.6 (1.5)	6.7 (1.4)	6.5 (1.5)	0.232
Sleep disturbance index (0–7), *n* = 272	1.7 (1.8)	1.4 (1.8)	1.9 (1.9)	0.004
Demographics				
Age (*n* = 276), mean (SD)	30.8 (5.8)	30.2 (5.9)	31.3 (5.7)	0.141
Race (*n* = 276), *n* (%)				<0.001
American Indian or Alaska Native	1 (0.3)	1 (0.8)	0 (0.0)	
Asian	4 (1.5)	0 (0)	4 (2.7)	
Native Hawaiian/Pacific Islander	3 (1.1)	2 (1.6)	1 (0.7)	
Black or African American	39 (14.1)	3 (2.4)	36 (24)	
White	151 (54.7)	51 (40.5)	100 (66.7)	
More than one race	12 (4.3)	3 (2.4)	9 (6)	
Prefer not to say	66 (23.9)	66 (52.4)	0 (0)	
Education (*n* = 276), *n* (%)				0.006
Some or less than high school	51 (18.5)	33 (26.2)	18 (12.0)	
High school diploma or GED	93 (33.7)	45 (35.7)	48 (32.0)	
Some college or technical school	72 (26.1)	25 (19.8)	47 (31.3)	
College degree or higher	60 (21.7)	23 (18.3)	37 (24.7)	
No. of children (*n* = 276), median [IQR]	2 [2]	2 [1]	2 [2]	0.078
Employment (*n* = 275), *n* (%)				
Not employed for wages, *n* (%)	167 (60.7)	97 (77.6)	70 (46.7)	<0.001
Assistance program participation (*n* = 275), *n* (%)				
SNAP	115 (41.7)	38 (30.2)	77 (51.3)	<0.001
WIC	193 (69.9)	103 (81.8)	90 (60.0)	<0.001
Other	15 (5.4)	6 (4.8)	9 (6.0)	0.651
Family history of chronic disease (*n* = 276), *n* (%)				
Diabetes	170 (61.6)	68 (54.0)	102 (68.0)	0.017
Hypertension	169 (61.2)	58 (46.0)	111 (74.0)	<0.001
Basic needs, *n* (%)				
Food insecure, *n* = 274	110 (40.2)	57 (45.6)	53 (35.6)	0.092
Basic needs met, *n* = 174	117 (67.2)	50 (69.4)	67 (65.7)	0.603
Stress				
PSS score (*n* = 276), mean (SD)	5.1 (3.3)	4.8 (3.2)	5.4 (3.4)	0.083
Physical activity category, *n* = 233				0.976
Low	115 (49.4)	50 (48.5)	65 (50.0)	
Moderate	58 (24.9)	26 (25.3)	32 (24.6)	
High	60 (25.7)	27 (26.2)	33 (25.4)	

BMI, body mass index; DBP, diastolic blood pressure; GED, General Education Development; IQR, interquartile range; PSS, perceived stress scale; SBP, systolic blood pressure; SD, standard deviation; SNAP, Supplementary Nutritional Assistance Program; WIC, Women, Infants, and Children.

### Differences between groups

BMI, systolic blood pressure, and diastolic blood pressure were significantly higher in the women identified as non-Hispanic/Latino as compared with women who identified as Hispanic/Latino (Table 1, *p* < 0.001). Similarly, family history of diabetes and hypertension were also more frequent in women who identified as non-Hispanic/Latino (Table 1, *p* < 0.05). Women who identified as Hispanic/Latino were more likely to report not being employed for wages outside of the home (77.6% vs. 46.7%, *p* < 0.001) and less likely to report completion of at least some college (*p* = 0.006) than women who identified as non-Hispanic/Latino.

Food insecurity was high in both groups (40% for the entire cohort), but the majority of women (117 [67.2%] for the entire cohort) reported that their basic needs were likely to be met. There were high rates of participation in SNAP or WIC in both groups, 81.8% of women who identified as Hispanic/Latino and 60.0% of women who identified as non-Hispanic/Latino participated in WIC and 30.2% of women who identified as Hispanic/Latino and 51.3% of women who identified as non-Hispanic/Latino participated in SNAP (Table 1).

Half of the women in both groups reported low levels of physical activity (49.4% for the entire cohort), and both groups self-reported <7 hours of sleep (6.6 [1.5] for the entire cohort). Women who identified as non-Hispanic/Latino reported higher levels of sleep disturbance as compared with women identifying as Hispanic/Latino (1.9 [1.9] vs. 1.4 [1.8], *p* = 0.004), but stress scores were similar between groups.

### Correlations of cardiometabolic variables with sleep, stress, basic needs, and demographics

#### Body mass index

In women who identified as Hispanic/Latino, BMI was significantly correlated with sleep disturbance (ρ = 0.23, *p* = 0.01, Table 2), but not sleep duration. In contrast, in women who identified as non-Hispanic/Latino, BMI was not significantly associated with sleep duration or sleep disturbance. Stress was not significantly associated with BMI in either group. In women who identified as non-Hispanic/Latino, BMI was significantly correlated with food insecurity (ρ = 0.17, *p* = 0.04, Table 3), and number of unmet basic needs (ρ = 0.25, *p* = 0.01, Table 3).

**Table 2.** Correlations among women identifying as Hispanic/Latino

	BMI	SBP	DBP	Sleep duration	Sleep disturbance	PSS score	IPAQ category
Cardiometabolic variables							
SBP	**0.33** ^a^						
DBP	**0.36** ^a^	**0.81** ^a^					
Sleep							
Sleep duration	−0.14	0.03	−0.10				
Sleep disturbance	**0.23**	−0.08	−0.02	−**0.57**			
Stress							
PSS score	0.06	−0.06	−0.09	−**0.20**	**0.29**		
Physical activity							
IPAQ category	−0.07	−0.03	−0.03	−**0.24**	0.04	0.14	
Demographics							
Education	−0.01	0.05	−0.13	−0.08	**0.21**	−0.003	−0.14
No. of children	0.02	0.14	**0.20**	**0.22**	−**0.23**	−0.003	−0.05
Employed	0.16	0.04	−0.08	−**0.25**	0.11	−0.09	0.05
Family history of diabetes	**0.24**	0.06	0.05	−**0.29**	**0.33**	0.10	−0.05
Family history of hypertension	**0.24**	**0.22**	**0.20**	−**0.31**	**0.30**	0.09	0.04
Basic needs							
Food insecure	0.11	−0.06	−0.02	0.07	−0.04	**0.28**	0.11
No. of unmet basic needs	−0.05	−0.01	−0.14	0.15	0.02	**0.30**	−0.07

Bold values indicate *p* < 0.05.

Pearson's correlations denoted by ^a^, otherwise values are Spearman's rho.

IPAQ, International Physical Activity Questionnaire; PSS, perceived stress score.

**Table 3.** Correlations among women identifying as non-Hispanic/Latino

	BMI	SBP	DBP	Sleep duration	Sleep disturbance	PSS score	IPAQ category
Cardiometabolic variables							
SBP	0.07^a^						
DBP	**0.20** ^a^	**0.77** ^a^					
Sleep							
Sleep duration	−0.08	−0.003	0.02				
Sleep disturbance	0.05	0.06	0.02	−**0.48**			
Stress							
PSS score	0.05	0.002	−0.10	−**0.24**	**0.35**		
Physical activity							
IPAQ category	−0.03	0.02	0.02	−0.11	0.04	−0.02	
Demographics							
Education	−0.02	0.04	0.08	−**0.17**	0.004	**0.16**	−0.03
No. of children	−0.02	0.03	0.04	−0.10	0.16	0.06	0.01
Employed	0.03	0.01	0.03	−0.01	−0.12	0.04	−0.09
Family history of diabetes	−0.001	0.10	0.02	−0.07	0.11	−0.01	0.02
Family history of hypertension	0.04	**0.22**	0.10	−0.05	**0.20**	0.04	−0.12
Basic needs							
Food insecure	**0.17**	−0.03	−0.06	−0.11	−0.03	**0.37**	0.04
No. of unmet basic needs	**0.25**	−0.03	−0.09	−0.01	0.07	**0.31**	0.15

Bold values indicate *p* < 0.05.

Pearson's correlations denoted by ^a^, otherwise values are Spearman's rho.

#### Blood pressure

Systolic and diastolic blood pressure were significantly related to BMI in both groups of women (*p* < 0.001 for all, Tables 2 and 3). Systolic blood pressure was related to family history of hypertension in both groups of women (*p* < 0.05 for both), whereas diastolic blood pressure was related to family history of hypertension (ρ = 0.20, *p* = 0.04, Table 2) in only women who identified as Hispanic/Latino.

### Correlations between sleep, stress, basic needs, physical activity, and demographics

Sleep duration was significantly negatively correlated and sleep disturbance positively correlated with stress in both groups (*p* < 0.05 for all, Tables 2 and 3). Neither sleep duration nor sleep disturbance were correlated with food insecurity or unmet basic needs in either group, but stress was positively correlated with both food insecurity and unmet basic needs in both groups (Tables 2 and 3).

Sleep duration was negatively correlated with employment (indicating women who were employed for wages outside the home slept shorter durations) (ρ = −0.25, *p* = 0.01, Table 2) and positively associated with number of children (ρ = 0.22, *p* = 0.01, Table 2) in women who identified as Hispanic/Latino, but there was no relationship between these variables in women who identified as non-Hispanic/Latino.

In women who identified as Hispanic/Latino, sleep disturbance was positively correlated and sleep duration negatively correlated with a family history of diabetes (ρ = 0.33, *p* = 0.0001, and ρ = −0.29, *p* = 0.002, respectively; Table 2) and hypertension (ρ = 0.30, *p* = 0.0001, and ρ = −0.31, *p* = 0.001, respectively; Table 2). Shorter sleep duration was correlated with higher physical activity category in women identifying as Hispanic/Latino (ρ = −0.24, *p* = 0.02), but not in women identifying as non-Hispanic/Latino. Higher physical activity was not correlated with stress or unmet basic needs in either group.

## Discussion

In this study, we found that women with overweight or obesity of childbearing age self-reported short sleep durations and some degree of sleep disruption. Women who identified as Hispanic/Latino reported less sleep disturbance compared with women who identified as non-Hispanic/Latino. In women who identified as Hispanic/Latino, sleep disturbance was related to increased BMI, but not in women who identified as non-Hispanic/Latino. Sleep and unmet basic needs were correlated with stress in both groups, but sleep and unmet basic needs were not correlated in either group.

Furthermore, sleep was related to multiple demographic characteristics (*i.e.*, education, number of children in the home, and employed for wages) and physical activity in women identifying as Hispanic/Latino, but these relationships were not seen in women identifying as non-Hispanic/Latino. Overall, our results suggest potential differences in factors that are related to sleep and cardiometabolic health in women who identify as Hispanic/Latino as compared with those who identify as non-Hispanic/Latino.

Our findings related to the relationship between stress, sleep, and unmet basic needs are consistent with previous research,^9,24–26^ but extend these findings by looking at the relationships specifically in women of childbearing age. Our findings are in contrast with Gaston et al.,^27^ who found that the relationship between short sleep and insomnia symptoms with metabolic syndrome did not differ by ethnicity in premenopausal women. However, our sample of women was younger (average ∼30 years compared with ∼47 years).

Poor sleep quality (*e.g.*, short duration and higher levels of sleep disturbance) has previously been reported in women of childbearing age.^1^ In our study, women who identify as Hispanic/Latino reported less disturbed sleep than women who identify as non-Hispanic/Latino. This is contrary to what some others have reported in other samples;^2,4,6,7,28^ however, more Hispanic/Latino people who were not born in the United States have reported getting the recommended 7 hours of sleep as compared with non-Hispanic White and Hispanic/Latino people born in the United States.^5^

Furthermore, in another study, sleep disparities were found to vary by birthplace and Hispanic/Latino heritage with Mexican adults less likely and Puerto Rican adults more likely to report short sleep duration as compared with non-Hispanic White adults.^29^ We did not ask women their birth country of origin in this study, but future larger studies should investigate if country of birth may impact sleep quality, particularly in women of childbearing age.

In our study, women identifying as Hispanic/Latino had lower BMI and blood pressure as compared with women identifying as non-Hispanic/Latino. Studies have shown that people who identify as Hispanic/Latino often tend to have better health outcomes (*e.g.*, lower rates of cardiovascular disease) as compared with their non-Hispanic/Latino White counterparts born in the United States,^2,30^ but there is an argument as to whether this phenomenon is fading.^31^

Interestingly, in this study, despite a lower BMI and lower self-reported sleep disturbance as compared with women who identify as non-Hispanic/Latino, BMI and sleep disturbance were significantly related in women identifying as Hispanic/Latino. Although there is a known connection between sleep disturbance and obesity,^32^ disparities in the relationship between sleep and obesity among racial and ethnic minorities have been reported, with racial and ethnic minorities experiencing disproportionately high rates of sleep disturbance and obesity.^2,28,33,34^

Gaston et al.^27^ reported that short sleep and insomnia symptoms were related to the metabolic syndrome in premenopausal and postmenopausal women; however, they found that the relationship varied by ethnicity only in postmenopausal, not premenopausal women. Our findings may differ due to age and ethnicity makeup of the studies. Our sample was younger with an average age of 30.8 years as compared with the average age of 46.8 for the premenopausal group, further only 5% of the sample identified as Hispanic/Latino as compared with 46% of our sample.^9,24–26^ Our findings suggest that decreasing sleep disturbance, particularly in women identifying as Hispanic/Latino, may be related to reduced obesity.

Sleep disturbance and sleep duration were correlated with higher stress levels in both groups of women in our study. Stress has been shown to be related to poor sleep previously^9^ and interestingly, acculturation stress in people who identify as Hispanic/Latino has been shown to be related to poorer sleep.^26^ We did not measure acculturation stress in this study, but future studies in women of childbearing age should investigate if acculturation stress plays a role in poorer sleep in women who identify as Hispanic/Latino. Stress was also related to higher unmet basic needs and food insecurity in both groups.

Our results extend previous findings that stress is related to both poor sleep quality and higher unmet basic needs.^9,11^ In this study, sleep was not directly related to unmet basic needs or food insecurity in either group; however, it may be possible that helping women to access resources and meet basic needs may indirectly benefit their sleep by reducing stress levels, as stress and sleep were correlated in both of the groups of women.

Furthermore, in women who identify as non-Hispanic/Latino, BMI was related to both unmet basic needs and food insecurity. Previous studies have shown this relationship,^24,25^ but our findings strengthen evidence to suggest that unmet basic needs in women of childbearing age are related to weight. It is unclear why this was not seen in women who identify as Hispanic/Latino, but it may be due to the lower sample size for the basic needs questions. Owing to the exploratory nature of the analysis, a multivariate regression analysis was not conducted, but these results can serve as a basis for future larger longitudinal investigation into this potential relationship.

In women who identified as Hispanic/Latino, sleep duration was negatively correlated with physical activity category, indicating that sleep duration was lower for people who reported higher levels of activity. Previous studies have found conflicting relationships between physical activity and sleep duration.^13,14^ Anecdotally, many people wake up early to exercise, which likely results in a decrease in sleep duration.

Sleep and physical activity are positively related to health,^35^ and higher physical activity has been shown to attenuate the relationship between short sleep time and mortality,^36^ but more studies are needed to determine if shortening sleep to exercise has beneficial effects on cardiovascular health in women of childbearing age. Demographic variables were also related to sleep duration and sleep disturbance, particularly in women identifying as Hispanic/Latino. Shorter sleep duration was associated with employment outside of the house. Although this is not surprising, it highlights that interventions aimed to improve sleep may need to be tailored to an individual's life circumstances.

Strengths of our study include an ability to compare relationships between cardiometabolic variables, sleep, stress, unmet basic needs, demographics, and physical activity among women of childbearing age who identify as Hispanic/Latino and among women who identify as non-Hispanic/Latino. Limitations of our study include possible residual confounding effects in the relationships observed. Owing to the small sample size, multivariable modeling was not performed, thus further study and replication of these findings are needed.

Other limitations include the cross-sectional nature of the data, sleep only measured as self-reported data and not objectively measured, and the small sample size for determining relationships of variables with unmet basic needs. Furthermore, the racial breakdown of our two groups was heterogenous (Table 1). Sleep disparities have been documented across races, particularly between non-Hispanic Black and non-Hispanic White individuals.^4,7^

Importantly, no differences in our results were seen when we removed all races except individuals who reported their race to be White in the non-Hispanic/Latino group (data not shown). However, the relationships that we found in this study could help to determine effect sizes for larger studies aimed at understanding the complex relationships among sleep, stress, and unmet basic needs with cardiometabolic variables.

## Conclusions

In summary, we found that sleep was related to BMI in women of childbearing age with obesity or overweight and who identify as Hispanic/Latino, whereas unmet basic needs and food insecurity were related to BMI in women who identify as non-Hispanic/Latino. Furthermore, we found that there were relationships between sleep and stress in both groups of women.

Stress was also related to unmet basic needs and food insecurity in both groups. Future studies are needed to confirm and extend these findings in larger groups with multivariate or mediation modeling. Overall, our results suggest that sleep, stress, and basic needs are important in understanding cardiometabolic health in women of childbearing age and these relationships differ depending on ethnicity.

## Abbreviations Used

BMI: body mass index
DBP: diastolic blood pressure
GED: General Education Development
IPAQ: International Physical Activity Questionnaire
IQR: interquartile range
PAT: Parents as Teachers
PSS: perceived stress scale
SBP: systolic blood pressure
SD: standard deviation
SNAP: Supplementary Nutritional Assistance Program
WIC: Women, Infants, and Children
